# Low birth weight in offspring of women with depressive and anxiety symptoms during pregnancy: results from a population based study in Bangladesh

**DOI:** 10.1186/1471-2458-10-515

**Published:** 2010-08-26

**Authors:** Hashima E Nasreen, Zarina Nahar Kabir, Yvonne Forsell, Maigun Edhborg

**Affiliations:** 1 Research and Evaluation Division, BRAC, 75 Mohakhali, Dhaka 1212, Bangladesh; 2 School of Public Health, BRAC, 66 Mohakhali, Dhaka 1212, Bangladesh; 3 Department of Neurobiology, Care Sciences and Society, Karolinska Institutet, SE-141 83 Huddinge, Stockholm, Sweden; 4 Department of Public Health, Karolinska Institutet, SE-17176 Stockholm, Sweden

## Abstract

**Background:**

There is a high prevalence of antepartum depression and low birth weight (LBW) in Bangladesh. In high- and low-income countries, prior evidence linking maternal depressive and anxiety symptoms with infant LBW is conflicting. There is no research on the association between maternal mental disorders and LBW in Bangladesh. This study aims to investigate the independent effect of maternal antepartum depressive and anxiety symptoms on infant LBW among women in a rural district of Bangladesh.

**Methods:**

A population-based sample of 720 pregnant women from two rural subdistricts was assessed for symptoms of antepartum depression, using the Edinburgh Postpartum Depression Scale (EPDS), and antepartum anxiety, using the State Trait Anxiety Inventory (STAI), and followed for 6-8 months postpartum. Infant birth weight of 583 (81%) singleton live babies born at term (≥37 weeks of pregnancy) was measured within 48 hours of delivery. Baseline data provided socioeconomic, anthropometric, reproductive, obstetric, and social support information. Trained female interviewers carried out structured interviews. Chi-square, Fisher's exact, and independent-sample *t *tests were done as descriptive statistics, and a multiple logistic regression model was used to identify predictors of LBW.

**Results:**

After adjusting for potential confounders, depressive (OR = 2.24; 95% CI 1.37-3.68) and anxiety (OR = 2.08; 95% CI 1.30-3.25) symptoms were significantly associated with LBW (≤2.5 kg). Poverty, maternal malnutrition, and support during pregnancy were also associated with LBW.

**Conclusions:**

This study provides evidence that maternal depressive and anxiety symptoms during pregnancy predict the LBW of newborns and replicates results found in other South Asian countries. Policies aimed at the detection and effective management of depressive and anxiety symptoms during pregnancy may reduce the burden on mothers and also act as an important measure in the prevention of LBW among offspring in Bangladesh.

## Background

Birth weight is generally used as a yardstick of intrauterine growth and as an important determinant of child survival and development [1]. Low birth weight (LBW) (<2500 g) remains a major problem in low-income countries affecting over 90% of the world's total infants [2]. LBW is associated with increased risk of infant mortality and morbidity [1]. In addition, there is an increased risk of neurodevelopmental outcome [3], cardiovascular disease [4], diabetes [5], emotional problems [6], and psychotic illness [7] in later life.

In past decades, there have been conjectures regarding the potential etiologic association of psychosocial factors, and particularly depressive symptoms, with LBW [8]. Conceptual models linking exposure to antepartum psychological stress have hypothesized on possible direct and indirect effects on LBW. There is some evidence supporting the direct effects of the psychoneuroendocrine process on poor neonatal outcome, especially birth weight [9,10]. Impaired mental health has also been associated with unhealthy maternal antenatal behavior including reduced attendance for antenatal care, increased substance use, and lower weight gain in pregnancy [11], which in turn has led to an increased likelihood of LBW [12]. Despite these vulnerabilities, the evidence linking maternal depressive and anxiety symptoms with infant LBW is conflicting. Studies from India [13], Pakistan [14], and Brazil [15] found an association between antepartum mental disorders and LBW. Studies from the United States [16], Sweden [17], China [18], and Ethiopia [19] have shown no significant association between LBW and maternal depressive symptoms. In high-income countries, positive associations between antepartum mental disorder and LBW have been reported in studies of disadvantaged populations [20] where socioeconomic status acts as an effect modifier. The comparability of study results is complicated, however, by the diversity of definitions, the measurement of prenatal maternal depressive symptoms, and the time points of assessment. The research in South Asia did not address the association between anxiety during pregnancy and LBW, but the strong correlation between anxiety and depressive measures suggests anxiety and depression should be examined concurrently [21]. In order to confirm the evidence from South Asia, the research needed to be replicated in other countries of South Asia, one of them being Bangladesh, where the estimated point prevalence of antepartum depression is as high as 33% [22] and LBW 36% [23].

Every year in Bangladesh, more than one million babies are born with LBW [24]. This causes great concern because of the strong association between LBW and child mortality and morbidity [24]. The neonatal mortality rate is 41-42 per thousand live births [25]. The determinants of the high prevalence of LBW are poorly understood. Determinants identified thus far are related to poverty, maternal nutritional status, and obstetric factors [23]. Despite substantial improvement in the poverty and health situation in Bangladesh, the state of LBW and neonatal mortality has remained static over the period. One explanation might be the recently reported high frequency of depressive symptoms in pregnant women. In this prospective community-based study, we addressed this shortcoming by examining the association between depressive and anxiety symptoms during the third trimester of pregnancy and LBW babies at term among rural women in Bangladesh.

## Methods

### Study setting

This is part of a prospective longitudinal study of perinatal depressive and anxiety symptoms among women in two subdistricts of the Mymensingh district (120 km north of the capital city, Dhaka) of Bangladesh. As is typical of rural Bangladesh, the economy in the study area is agrarian, and approximately 50% of the population lives below the poverty level. The majority of women are involved in household work and childcare. A national nongovernment development organization, the Bangladesh Rural Advancement Committee (BRAC) provides a variety of services in the area for social and economic development. The BRAC health program provides preventive health and nutritional education, immunization, family planning, pregnancy and reproductive-health care, and basic curative services. BRAC community health volunteers identify pregnancies during the first trimester, estimate the gestational age (based on the last menstrual period reported by the women), confirm pregnancies at 4-5 months, and register them.

### Study design and populations

The pregnancy registration system maintained by BRAC provided the sampling frame. The gestational age recorded in the register was verified by the interviewers during data collection. A cohort of 720 consecutive women was studied from the third trimester of pregnancy to 6-8 months postpartum. With an average population of 1250 persons per village in Bangladesh and a delivery rate of 3%, 37 women were expected to give birth in each village per year. Therefore, 154 villages were needed from 10 randomly selected unions to obtain the required sample. Assuming an estimated prevalence of depression of 20% in India [26] and Pakistan [27] (no prevalence figure was available for Bangladesh when the study was initiated), the study was designed with a precision of 0.05, power of 0.80, and an effect size of 0.40 to detect the difference between depressed and non-depressed women.

**Figure 1:**
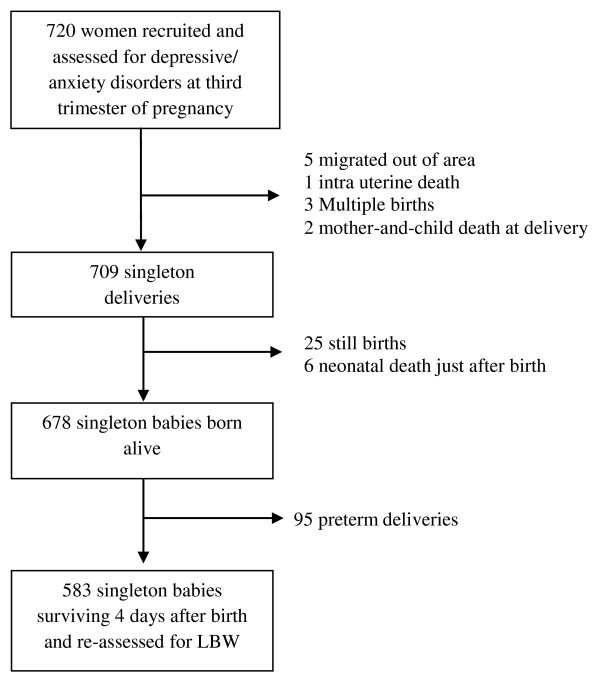
**Sample profile**.

The exclusion criteria for the original study were emigration from the study area, intrauterine death, or abortion. In the present study, 583 (81%) singletons, live births, and infants born at term (≥37 weeks of pregnancy) were included (Figure 1). The reason for the inclusion of only singletons was that infants from multiple births are known to be at greater risk of preterm birth and retardation of fetal growth [28].

### Data collection

Data was collected from July 2008 to August 2009. Data at baseline (third trimester pregnancy) included socioeconomic conditions and the women's anthropometric status, reproductive health, social support, exposure to violence, anxiety, and depressive symptoms. Birth data were collected through structured interviews by trained female interviewers in the homes of the women upon their recovery from labor as a majority of deliveries (85%) occurred at home. The time periods varied between 2 to 48 hours following delivery. In hospital delivery cases, birth data were taken from the hospital records. A broad array of obstetric outcomes was assessed: length of pregnancy, mode and place of delivery, complications during labor, live or still birth, and birth weight, height, and head circumference of the newborn.

### Measurements

#### Assessment of antepartum depressive symptoms

The Edinburgh Postnatal Depressive Scale (EPDS) [29] was used to detect depressive symptoms during pregnancy. The EPDS is a 10-item questionnaire, scored from 0 to 3 (normal response 0 and severe response 3), that has been validated for the detection of depression in antepartum and postpartum samples in many countries [30]. The instrument has been validated for Bangladesh, which showed a sensitivity of 89%, a specificity of 87%, a positive predictive value of 40%, and a negative predictive value of 99% [31]. The cutoff score suggested by Gausia [31] was used to categorize depressed (score ≥10) and non-depressed (score <10) states. The scale shows good reliability in the present study with a Cronbach's alpha of 0.87 for the assessment of antepartum depressive symptoms.

#### Assessment of antepartum anxiety symptoms

From the State Trait Anxiety Inventory (STAI) [32] we used the trait-anxiety scale consisting of 20 items scored from 1 to 4. STAI assesses anxiety levels in general during pregnancy in feelings of pleasure, nervousness, restlessness, satisfaction, happiness, and so on. STAI is a reliable and valid measure that can be used in both clinical and general populations [32]. The cutoff score of 46 (75th percentile) was used to categorize anxious (score ≥46) or non-anxious (score <46) states. STAI demonstrated good internal consistency in the present study with a Cronbach's alpha of 0.82 for antenatal assessment.

#### Assessment of socioeconomic, anthropometric, reproductive, obstetric, and social support status

The age of the mother was calculated in years. Socioeconomic status was indicated by parental education (years of completed schooling) and the economic status of the household. Two measures were used to assess household economy: landholding of the household and per capita daily household expenditure on food. Poor household economic status was assessed if the household owned <50 decimals of land or if the per capita daily household expenditure on food was less than the median in Bangladeshi taka of BDT 31.25 (USD 0.45). The anthropometric indicators of the pregnant women used in the study were mean weight (kg), mean maternal body mass index (BMI) [weight (kg)/height (m)^2^], and mean mid upper arm circumference (MUAC) (cm). The precise position of the arm measurement was at the midpoint between the tip of the acromion and the olecranon processes in the left upper arm to the nearest 0.1 cm. BMI was measured during pregnancy, however, so we must assume it to be over-reported; thus, MUAC was seen as a proxy indicator of the nutritional status of the women. Reproductive indicators included the number of children and antenatal consultations provided by health personnel. Social support was measured by family structure, such as living in a nuclear family or extended family, physical and psychological support (coming from family members, friends, or health professionals), and physical violence (being slapped/dragged/subjected to thrown objects) at any time in life or during pregnancy.

For the purposes of analysis, the explanatory variables were dichotomized. Maternal age was expressed as ≤20 years vs. 21 years or older; parental education as ≤5 years of education of each parent vs. >5 years of education; household economic status as poor vs. nonpoor; MUAC as <22 cm vs. ≥22 cm [33]; number of children as ≥4 children vs. <4 children (families with ≥4 children in low-income countries were assumed to cause financial crisis and overcrowding); antenatal consultation as having been provided at least one vs. none; family structure as living in a nuclear family vs. living in an extended family; physical and psychological support as support received vs. no support received during pregnancy; and physical violence experienced as yes vs. no.

#### Assessment of obstetric outcome

Obstetric outcome was measured during delivery in terms of complicated labor (if any complication arose during delivery) and mode of delivery (instrumental by cesarean section or normal vaginal delivery), birth weight (kg), and head circumference (mm) of the newborn. We did not consider smaller head circumferences (<2 standard deviation of mean) as a dependent variable as it was strongly correlated with LBW (r = 0.560, p = 0.000).

#### Assessment of birth weight

Infant birth weight was measured to the nearest 0.1 kg within 48 hours of birth by trained interviewers using a portable digital Salter bathroom scale (Japan). The mother was requested to hold the baby while being weighed, and the baby's weight was calculated by subtracting the mother's weight from the sum weight of mother and baby. The standard cutoff for LBW is 2500 grams or less [1], and this was termed the dependent variable.

### Statistical analysis

We first compared depressed vs. non-depressed and anxious vs. non-anxious women by their socioeconomic, anthropometric, obstetric, and pregnancy outcome indicators using chi-square or Fisher's exact test. An independent samples *t *test was used to compare means between depressed vs. non-depressed and anxious vs. non-anxious groups. Univariate logistic regression analyses were carried out to identify possible predictors with a 95% confidence interval (p < 0.05) of being associated with LBW (≤2500 g). Adjusted odds ratios for all variables that were significantly associated with LBW were computed using a multiple logistic regression model for controlling the simultaneous confounding effects of possible predictors. Model I shows the role of antepartum depressive symptoms and Model II shows anxiety symptoms as predictors of LBW. Any violations of the assumptions were observed by examining the interaction between explanatory variables and outliers in the model.

### Ethical considerations

The study was approved by the Bangladesh Medical Research Council (Ref. no. BMRC/Eth. C/2008/402) in Bangladesh and the Regional Ethical Review Board at Karolinska Institutet, Stockholm, Sweden (Ref. no. 2008/919-31). Detailed information about the study was provided verbally to the potential participants. The interviews were conducted after informed consent was obtained. Strict confidentiality was maintained about the identity of the respondents. If a woman scored more than16 on the EPDS during the study, we advised her to consult the psychiatric department of the nearby Mymensingh Medical College Hospital.

## Results

The prevalence of depressive symptoms was 132 (18%) and general anxiety was 186 (26%) among all women (N = 720) in the last trimester of pregnancy. None of the women who suffered from depressive symptoms sought help from the qualified practitioners and used any antidepressants (not shown). Of the 583 mothers in the present study, 107 (18%) were identified as experiencing depressive symptoms (mean score 12.3 ± 2.8) and 149 (26%) general anxiety during the last trimester of pregnancy (mean score 50.2 ± 4.6). Analysis revealed that depressed and anxious women were older, less educated, and had a lower body weight than non-depressed and non-anxious women. Anxious women were more likely to be poor in terms of landholding and per capita daily household expenditure on food, and had a lower BMI and MUAC than the non-anxious women. No significant differences were noted with respect to complicated labor and instrumental delivery (Table 1).

**Table 1:** Description of the study sample by maternal antepartum depression and anxiety status (N = 583)

	Depressed N = 107	Non-depressed N = 476	*p*	Anxious N = 149	Non-anxious N = 434	*p*
**Socioeconomic**						
Mean age ± SD	26.7 ± 6.9	24.1 ± 5.7	0.000	25.4 ± 6.4	24.3 ± 5.9	0.041
Mean years of schooling ± SD	3.01 ± 3.3	3.9 ± 3.6	0.021	2.8 ± 3.2	4.0 ± 3.6	0.000
Land (<50 decimal) (%)	72 (67.3)	284 (59.7)	0.144	103 (69.1)	253 (58.3)	0.019
Per capita daily household expenditure on food (<median*) (%)	61 (57.0)	232 (48.7)	0.122	93 (62.4)	200 (46.1)	0.001

**Anthropometric**						
Weight ± SD (kg)	46.3 ± 5.6	48.3 ± 6.9	0.004	46.4 ± 5.7	48.5 ± 7.1	0.001
BMI ± SD	21.0 ± 2.1	21.4 ± 2.7	0.247	20.9 ± 2.0	21.4 ± 2.8	0.038
MUAC ± SD (cm)	23.1 ± 1.9	23.4 ± 2.4	0.191	22.9 ± 2.0	23.1 ± 2.4	0.036

**Obstetric**						
Complicated labor (%)	12 (11.2)	52 (10.9)	0.931	16 (10.7)	48 (11.1)	0.914
Instrumental delivery (%)	3 (2.8)	33 (6.9)	0.109	5 (3.4)	31 (7.1)	0.098

**EPDS mean score (± SD)**	12.3 ± 2.8	5.0 ± 2.4	0.000	-	-	

**STAI mean score (± SD)**	-	-		50.2 ± 4.6	37.8 ± 4.6	0.000

*Cutoff defined as median, or BDT 31.25 (USD 1 = BDT 69.13).

Table 2 illustrates pregnancy outcomes by antepartum depressive and anxiety symptoms. Irrespective of depression and anxiety status, 19% (n = 108) of the mothers had delivered LBW babies (not shown). The rate was higher among depressed and anxious women. No significant difference was found between depressed and non-depressed women, and anxious and non-anxious women, in terms of the average birth weight of children born to them and gestational age at delivery. Head circumference of the newborns was found to be significantly lower among the depressed and anxious women compared to their counterparts. There was no significant difference in preterm delivery (n = 95) and stillbirths (n = 25) among depressed vs. non-derpessed and anxious vs. non-anxious women (not shown).

**Table 2:** Birth outcome by maternal antepartum depressive and anxiety symptoms (N = 583)

Outcome	Depressed N = 107	Non-depressed N = 476	*p*	Anxious N = 149	Non-anxious N = 434	*p*
LBW (≤2.5 kg) (%)	33 (30.8)	75 (15.8)	0.000	43 (28.9)	65 (15.0)	0.000

Infant birth weight (kg, mean ± SD)	2.8 ± 0.5	2.9 ± 0.4	0.145	2.8 ± 0.5	2.9 ± 0.4	0.090

Gestational age at delivery (weeks, mean ± SD)	40.1 ± 1.5	40.0 ± 1.3	0.521	40.1 ± 1.4	40.0 ± 1.3	0.462

Head circumference (mm, mean ± SD)	33.7 ± 1.5	34.1 ± 1.4	0.029	33.8 ± 1.5	34.1 ± 1.4	0.020

Table values are mean and ± SD for continuous variables and n (%) for categorical variables.

Univariate regression analysis showed a significant positive association between LBW and antepartum depressive symptoms, antepartum anxiety symptoms, poor household economic status, and maternal malnutrition, and a significant negative association with maternal antepartum consultation, psychological support during pregnancy, and living in a joint family (Table 3).

**Table 3:** Association of newborn low birth weight with maternal antepartum depressive/anxiety symptoms and other factors (N = 583)

	Normal birth weight n = 475 (%)	Low birth weight n = 108 (%)	Unadjusted OR (95% CI)	*p*
Antepartum depressive symptoms	74 (15.6)	33 (30.6)	2.38 (1.47-3.84)	<0.001

Antepartum anxiety symptoms	106 (22.3)	43 (39.8)	2.30 (1.48-3.58)	<0.001

Mother's age ≤20 years	99 (20.8)	25 (23.1)	1.14 (0.69-1.88)	0.597

Mother low/uneducated	336 (70.7)	84 (77.8)	1.45 (0.88-2.38)	0.141

Land (<50 decimal)	275 (57.9)	81 (75.0)	2.18 (1.36-3.50)	0.001

Per capita food expenditure (<Md BDT.31.25)	234 (49.3)	59 (54.6)	1.24 (0.82-1.89)	0.314

Father low/uneducated	367 (77.4)	88 (81.5)	1.28 (0.75-2.18)	0.357

Maternal malnutrition (mother's MUAC <22 cm)	134 (28.2)	48 (44.4)	2.04 (1.33-3.13)	0.001

Being firstborn	136 (28.6)	24 (22.2)	0.71 (0.43-1.17)	0.178

Four or more siblings	88 (18.5)	21 (19.4)	1.06 (0.63-1.80)	.0.825

At least one antenatal consultation	220 (46.3)	37 (34.3)	0.60 (0.39-0.94)	0.023

Psychological support during pregnancy	462 (97.3)	97 (89.8)	0.25 (0.11-0.57)	0.002

Physical support during pregnancy	380 (80.0)	86 (79.6)	0.98 (0.58-1.64)	0.931

Living in a joint family	396 (83.4)	79 (73.1)	0.54 (0.33-0.89)	0.014

Physical violence: slapped, dragged, or subjected to thrown objects	327 (68.8)	84 (77.8)	1.58 (0.97-2.60)	0.066

Physical violence during pregnancy	87 (18.3)	20 (18.5)	1.01 (0.59-1.74)	0.961

Multiple logistic regression analyses (Table 4) showed that, after simultaneous adjustment for the associated factors, mothers with symptoms of depression (OR = 2.24; 95% CI 1.37-3.68) and anxiety (OR = 2.08; 95% CI 1.30-3.25) were twice as likely to give birth to LBW babies than mothers who did not report these symptoms. Other positively associated factors were poor economic status of household and maternal malnutrition; psychological support during pregnancy and living in a joint family (anxiety only) were negatively associated with LBW. A Wald estimate indicates that depressive and anxiety symptoms contributed most to LBW, followed by maternal malnutrition, emotional support during pregnancy, and poor household economic status. No significant interaction between explanatory variables was found. The models indicated almost the same Log likelihood ratio (-520.56 for Model I and -516.60 for Model II) after excluding the outliers (Cook's distance >0.049). A Hosmer-Lemeshow test indicated that Model I (p = 0.809) and Model II (p = 0.106) fit the data well.

**Table 4:** Final logistic regression model of depressive and anxiety symptoms during pregnancy on LBW

	Model I				Model II			
	**OR**	**SE**	**95% CI**	**Wald**	**OR**	**SE**	**95% CI**	**Wald**

Poor household economic status (land <50 decimal)	1.86	0.248	1.14-3.02	6.24	1.76	0.249	1.07-2.85	5.17

Maternal malnutrition (mother's MUAC <22 cm)	1.98	0.227	1.27-3.08	8.99	1.92	0.228	1.2-3.00	8.20

Emotional support during pregnancy	0.27	0.442	0.11-0.65	8.67	0.27	0.442	0.12-0.65	8.65

Joint family	-	-	-	-	0.59	0.259	0.36-0.98	4.12

Depressive symptoms during pregnancy	2.24	0.252	1.37-3.68	10.27				

Anxiety symptoms during pregnancy	-	-	-	-	2.08	0.233	1.32-3.29	9.92

## Discussion

This study revealed that women with depressive and anxiety symptoms in the third trimester of pregnancy exhibit an increased likelihood of giving birth to LBW infants in Bangladesh. This association is independent of the effects of poverty, maternal nutritional status, and support during pregnancy. This is consistent with previous research from other South Asian countries documenting that women who exhibit elevated depressive symptoms during pregnancy are at increased risk for delivering LBW infants [13,14]. Evidence from high-income countries is mixed, with negative associations in Sweden [17], Norway [6], and the United States [16]; a positive association may be apparent only under circumstances of socioeconomic adversity in the United States [20]. Negative association is also reported from sub-Saharan Africa [19] where the prevalence of LBW is as high as in the South Asian region. However, it is too early to determine whether there is an etiological heterogeneity across these settings because of the different cultures, health-care systems, and maternal and child health profiles. As none of the depressed women used any antidepressants in the third trimester, the antidepressants are unlikely to have any impact on LBW in this study.

The high prevalence of LBW (24%-36%) in Bangladesh [23,34,35] is one of the main causes of infant morbidity and mortality, and many studies have shown maternal nutrition to be an important predictor of LBW in low-income countries [1,36,37]. This study shows that maternal antepartum depression and anxiety are independent predictors of LBW irrespective of poor maternal nutritional status. Poor maternal nutritional status, the principal cause of LBW in low-income countries [38], is not necessarily a result of poverty but of maternal mental disorders such as antepartum depression and anxiety, even in the food-sufficient regions of rural Bangladesh. Similar situations have been observed in Pakistan [14], and the current study provides further evidence for the "Asian enigma" referred to in Rahman et al. [14].

Hoffman and Hatch [39] pointed out a possible association between antepartum depressive symptoms at 28 weeks of gestation and retardation of fetal growth among women of disadvantaged social groups, raising questions as to whether having a poorer socioeconomic status is a vulnerable factor per se. This study found that poverty, indicated here by economic status of household, is a potential explanatory variable of LBW. In impoverished communities, poverty is assumed to play the major role in determining LBW, associating low income with inadequate antenatal care and lower antenatal maternal weight [1].

Our finding of an independent and negative association between support during pregnancy (through joint family structure and psychological support) and the birth weight of the infant is a new result in a low-income South Asian country. It may be speculated that support during pregnancy alters the stress-induced hypothalamic-pituitary axis [40] that suppresses maternal cortisol levels, thereby restoring fetal automatic nervous system activities, reducing vascular constriction, and potentiating the uterine artery blood flow that carries oxygen and nutrients to the fetus. Previous studies by Lee et al. [41] and Hodnett and Frederick [42] on the impact of social support have shown conflicting results. Lee et al. [41] hypothesized that this kind of support helps the disadvantaged women by empowering them and improving their ability to be more engaged in self-care and antenatal care.

In South Asian countries, including Bangladesh, women are exposed to various socioeconomic, social, and family life stressors, which contribute significantly to maternal depressive and anxiety symptoms [22,43]. A women's life-course perspective proposes that the life stressors are not only linked with mental health disorders [44], but also to poor birth outcome and particularly LBW. Infants are likely to continue the cycle by being stunted in adulthood through cumulative pathways. This cumulative mechanism posits that stressors accumulating at different stages in the life of a woman lead to intrauterine growth retardation and LBW in her newborn, which in turn may lead to impaired mental development in infanthood, reduced intellectual potentials in childhood, depression in adolescence, and mental disorders in adulthood and later life [45].

This study has a number of strengths, including a community-based population from a defined geographical rural area in Bangladesh, a prospective design with minimal loss to follow-up, the measurement of maternal depression using a locally validated EPDS, and an analysis restricted to babies born at term to distinguish the risk factors of intrauterine growth restriction from those of preterm births. This restriction of the final sample to full-term deliveries may have resulted in the lack of difference in gestational age between depressed/anxious women and non-depressed/non-anxious women. The study was conducted in two subdistricts of rural Bangladesh and does not represent the urban scenario. Although the findings cannot be generalized even to other rural areas of the country, the community-based sample is likely to be indicative of the situation among rural women. Limitations of the study include the inability to control for several important variables such as anemia, weight gain during pregnancy, physical ill-health (diabetes/hypertension), and smoking (although smoking was uncommon among the women of our study population).

## Conclusions

This population-based study in rural Bangladesh found an independent association between maternal depressive and anxiety symptoms in the third trimester of pregnancy and infant LBW over and above the well-established risk factors of poverty and maternal malnutrition. The pattern of exposure associated with LBW in the final, adjusted model largely accords with that reported from the other South Asian countries. The reduction of LBW at term is an important indicator of the internationally agreed Millennium Development Goals for reducing child mortality and is a key indicator of progress. Our study indicates that, in order to achieve this goal, maternal depressive and anxiety symptoms during pregnancy need to be addressed. Policies aimed at the detection and effective management of depressive and anxiety symptoms during pregnancy cannot only reduce the burden on mothers but is an important preventive action for both LBW and the physical and mental health of offspring.

## Abbreviations

LBW: low birth weight; BRAC: Bangladesh Rural Advancement Committee; EPDS: Edinburgh Postnatal Depressive Scale; STAI: State Trait Anxiety Inventory; BMI: body mass index; MUAC: mid upper arm circumference

## Competing interests

The authors declare that they have no competing interests.

## Authors' contributions

All authors (HEN, ZNK, YF, ME) participated in the planning and conception of the research questions and the study design. ME was the principal investigator of the study and primarily conceptualized the research. HEN was responsible for retrieving the data, and HEN and ME were responsible for analyzing the data. HEN drafted the article, and all authors participated in interpreting the data and critically revising the manuscript for important intellectual content. All authors read and approved the revised manuscript.

## Pre-publication history

The pre-publication history for this paper can be accessed here:

http://www.biomedcentral.com/1471-2458/10/515/prepub
